# Antibacterial and Anti-Acne Activity of Benzoyl Peroxide Nanoparticles Incorporated in Lemongrass Oil Nanoemulgel

**DOI:** 10.3390/gels9030186

**Published:** 2023-02-27

**Authors:** Ahmad M. Eid, Hani Naseef, Nidal Jaradat, Lina Ghanim, Roaa Moqadeh, Miasar Yaseen

**Affiliations:** 1 Department of Pharmacy, Faculty of Medicine and Health Sciences, An-Najah National University, Nablus P.O. Box 7, Palestine; 2 Pharmacy Department, Faculty of Pharmacy, Nursing and Health Professions, Birzeit University, Ramallah P.O. Box 14, Palestine

**Keywords:** Benzoyl peroxide, nanoemulgel, lemongrass oil, self-emulsifying system, antibacterial activity

## Abstract

Purpose: The goal of this study was to make Benzoyl Peroxide (BPO) nanoemulgel to improve its ability to kill bacteria. BPO has trouble getting into the skin, being absorbed by the skin, staying stable, and being spread out. Methods: A BPO nanoemulgel formulation was prepared by combining BPO nanoemulsion with Carbopol hydrogel. The drug was tested for solubility in various oils and surfactants in order to select the best oil and surfactant for the drug, and then the drug nanoemulsion formulation was prepared using a self-nano-emulsifying technique with Tween 80, Span 80, and lemongrass oil. The drug nanoemulgel was looked at in terms of its particle size, polydispersity index (PDI), rheological behavior, drug release, and antimicrobial activity. Results: Based on the solubility test results, lemongrass oil was the best solubilizing oil for drugs, while Tween 80 and Span 80 demonstrated the highest solubilizing ability for drugs among the surfactants. The optimum self-nano-emulsifying formulation had particle sizes of less than 200 nm and a PDI of close to zero. The results showed that incorporating the SNEDDS formulation of the drug with Carbopol at various concentrations did not cause a significant change in the particle size and PDI of the drug. The zeta potential results for drug nanoemulgel were negative, with more than 30 mV. All nanoemulgel formulations exhibited pseudo-plastic behavior, with 0.4% Carbopol exhibiting the highest release pattern. The drug nanoemulgel formulation worked better against bacteria and acne than the product on the market. Conclusion: Nanoemulgel is a promising way to deliver BPO because it makes the drug more stable and increases its ability to kill bacteria.

## 1. Introduction

Nanotechnology can be defined as a technique through which the material is manipulated on a nano-scale form to get properties not found in the bulk form. Nanoparticles could be from natural sources such as viruses and allergens or created in labs [1,2]. This science plays an important role in medicine by producing drugs with better bioactivity, and this provides an opportunity to find new cures for different diseases [3,4]. A nanoemulsion is a drug delivery system that contains droplets ranging in diameter from 100 to 500 nm on average. This small size of particles provides a large surface area, which enhances absorption. In addition to that, it will reduce the coalescence and precipitation of emulsions [5].

The nanoemulsification technique is the result of numerous adjustments and enhancements to the drug delivery procedures’ limiting pathways. The phrase is the result of the combination of nanoemulsion and hydrogel. By reducing the surface and interfacial tension and increasing the viscosity of the aqueous, it is possible to improve the stability of a nanoemulsion due to its gelling system, as well as its topical administration. In addition, nanoemulgel, which is essentially gel, exhibits improved thixotropic qualities, is non-greasy, readily spreadable, simple to remove, does not stain, is water-soluble, emollient, has a longer shelf life and a pleasing look [6,7]. Since the formulation is not sticky, it eliminates the difficulty of spreading and promotes patient compliance during administration.

The second-leading cause of death worldwide, antimicrobial resistance is seen as a significant public health issue. In recent years, the World Health Organization (WHO) has expressed grave worry over the increase in antimicrobial resistance rates [8]. This necessitates new strategies for either the development of new antibiotics or the improvement of the efficacy of existing antibiotics. Consequently, a number of studies offer an overview of the management of microbial infection and antibiotic resistance. Nanostructured materials are one of the solutions being investigated to combat bacterial resistance [9]. Nanoparticles may therefore offer a promising answer as they can not only battle bacteria but also act as carriers for antibiotics by circumventing drug resistance mechanisms in bacteria and facilitating the administration of innovative medications [10,11].

Acne is an inflammatory skin disease caused by an anaerobic gram-positive bacteria called Propionibacterium, which causes inflammation and an increase in sebum production. It can also cause keratinization at the site of acne [4]. Acne affects 20% of teenagers’ skin and causes facial scarring, which is annoying [12]. Treatment of acne can control and help heal inflammation and scars. The treatment includes topical medications that can control mild to moderate acne, like a combination of Benzoyl Peroxide (BPO) and an antibiotic (Clindamycin) or retinoid [13]. Also, oral medications can be used, such as oral antibiotics and Isotretinoin for severe cases, in addition to topical medications [13,14,15].

BPO is a bactericidal agent and is widely used in the treatment of acne vulgaris [16,17]. BPO has keratolytic and anti-inflammatory effects on acne. It has a role in the production of highly reactive oxygen radicals, so it is an alternative to antibiotics that have become resistant to Propionibacterium acne [18,19,20]. BPO is very effective as a monotherapy for mild to moderate acne cases [20]. It is available on the market alone or in combination with other drugs, such as gels and creams [21,22]. The goal of this research is to reduce these side effects and improve the absorption, penetration, stability, and spreadability of the drug by developing a new formulation of BPO in the form of a nanoemulgel.

## 2. Results

### 2.1. Determination of the Optimum Wavelength and Calibration Curve of Benzoyl Peroxide

The test was carried out for BPO using a UV spectrophotometer to find the most appropriate and suitable wavelength that has the best absorbance. The highest absorbance was carried at 245 nm.

### 2.2. Solubility Testing of Benzoyl Peroxide in Different Oils and Surfactants

The dissolving process of BPO in different oils and surfactants helps us choose the best ones that have the highest solubility by using a UV spectrophotometer to determine the absorption. The results we got are shown in Table 1. Depending on the data shown in Table 1, the best solubilizing oil was lemongrass oil. In addition, the best solubilizing surfactants were Span 80 and Tween 80.

### 2.3. Optimization of Lemongrass Oil Nanoemulsion Formulation

**Figure 1:**
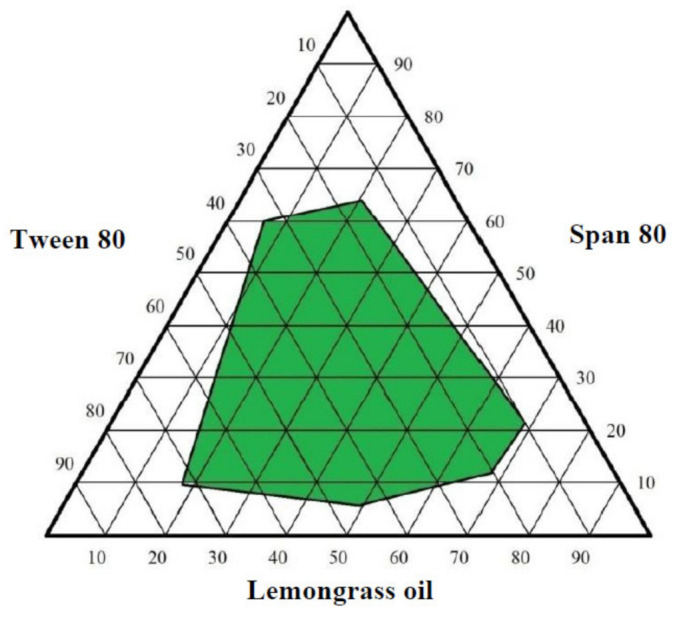
Pseudo ternary phase diagram of lemongrass oil, Tween 80 and Span 80 nanoemulsion.

Lemongrass oil, Span 80, and Tween 80 were mixed together in different concentrations in order to choose the optimum nanoemulsion formulation based on the ternary phase diagram. As presented in Figure 1, the ternary phase diagram showed that the formulation, which contained (30% of Tween 80, 10% of Span 80, and 60% Lemongrass oil), had a droplet size below 200 nm (185.47 ± 1.21 nm) and a size distribution below 0.3 (0.163 ± 0.07).

### 2.4. Particle Size, Polydispersity Index, and Zeta Potential of Benzoyl Peroxide Nanoemulgel

**Figure 2:**
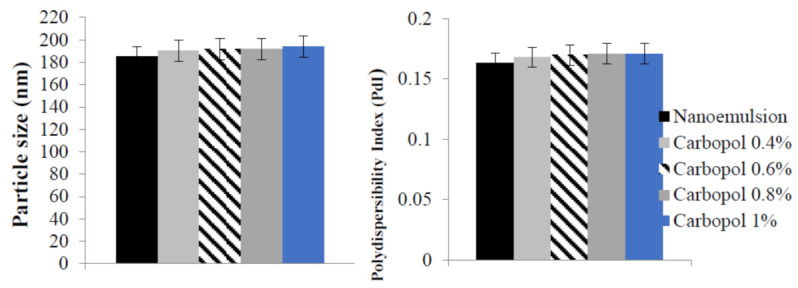
The particle size and polydispersity index (PDI) of Benzoyl peroxide nanoemulgel with different Carbopol concentrations.

**Figure 3:**
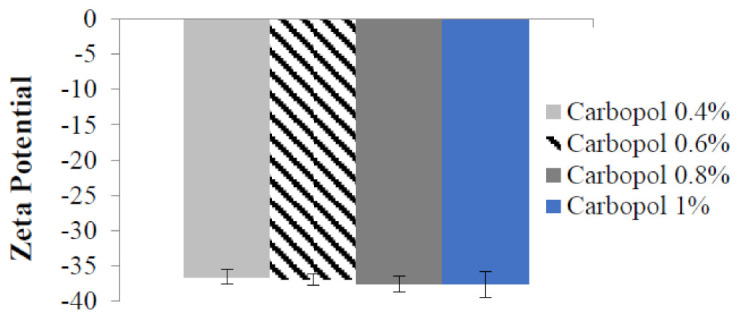
The zeta potential of Benzoyl peroxide nanoemulgel with different Carbopol concentrations.

After loading the nanoemulsion with the drug, BPO nanoemulgels were prepared. The mixture was then mixed with various concentrations of Carbopol hydrogel and water (0.4%, 0.6%, 0.8%, and 1%). Figure 2 and Figure 3 show the drug nanoemulgel particle size, PDI, and zeta potential results, respectively. In addition, the results were the same regardless of the concentration of Carbopol; the only variation was that a slight increase in particle size was seen in response to the rise in Carbopol concentration. Regarding the drug’s zeta potential, each of the nanoemulgel formulations had a value that was lower than −30 mV, and all of them were considered successful.

### 2.5. Rheological Behavior of Benzoyl Peroxide Nanoemulgel

**Figure 4:**
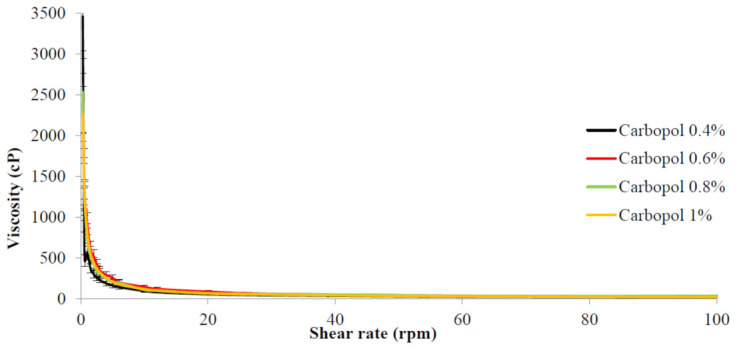
Rheological behavior of Benzoyl peroxide nanoemulgel prepared using different Carbopol concentrations.

The rheological characteristics, also known as flow properties, of drug nanoemulgel formulations, which are believed to be semi-solid, require to be assessed since these features might reflect and signal the formulation’s quality and effectiveness. The rheological investigation of the drug nanoemulgel formulation indicated that viscosity reduced as the shear rate rose, which indicates that the drug nanoemulgel formulation behaves as a pseudo-plastic substance (Figure 4). As shown in Figure 4, the Carbopol concentration had no effect on the behavior of nanoemulgel formulations. However, the only change was in the viscosity, which increased as the Carbopol concentration was increased.

### 2.6. Release Test of Benzoyl Peroxide from the Nanoemulgel Formulation

**Figure 5:**
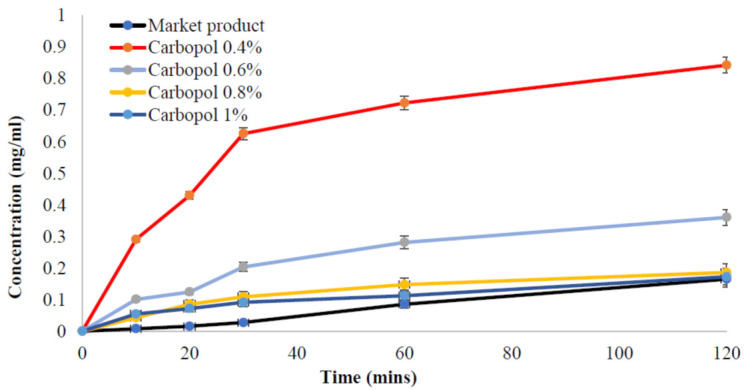
The release profile of Benzoyl peroxide nanoemulgels containing different Carbopol concentrations compared with the market product.

In order to study the release of the drug from the nanoemulgel formulations, tests were conducted. The results of these tests helped in the selection of the optimal concentration of Carbopol to be utilized as a thickening agent in the production of the hydrogel that was used in the nanoemulgel formulations. The release from the nanoemulgel formulations was compared to the release from the product on the market. Dialysis was the approach that was used in order to complete this objective. Figure 5 illustrates the many ways in which drugs unleash their effects on the body. It was observed that raising the concentration of Carbopol resulted in a decrease in the release profile, with the maximum drug release occurring at a Carbopol concentration of 0.4 percent. Additionally, as compared to the product currently available on the market, each of the nanoemulgel formulations had a greater release profile.

### 2.7. Antibacterial and Anti-Acne Evaluation of Benzoyl Peroxide Nanoemulgel

The antibacterial activity of the BPO nanoemulgel was tested on Methicillin-Resistant *Staphylococcus aureus* (MRSA), *Pseudomonas aeruginosa, E. coli*, *Klebsiella pneumonia*, *Staphylococcus aureus*, *Proteus mirabilis*, Candida and *Cutibacteruim* acne by implanting them in agar media on Petri dishes and comparing the inhibition zone to the market. The antibacterial activity is explained in Table 2, and the antibacterial acne activity is in Table 3.

## 3. Discussion

In this study, the nanoemulgel formulation of BPO helps in the improvement of drug penetration and absorption through the skin, minimizing side effects and increasing therapeutic activity compared with market products. This was achieved by combining the drug with Carbopol hydrogel after preparing it as a nanoemulsion using a self-nano emulsifying technique. The drug nanoemulgel and market products were subjected to drug release and antibacterial tests. To make a nanoemulsion using a self-emulsification method that has the best physiochemical properties, the active or inactive ingredient of the formula must be carefully chosen to have a good mix of oil, surfactant, and co-surfactant.

By analyzing the solubility of the drug, it is possible to discover self-nano emulsification system components with an adequate solubilizing ability for BPO. It is essential to test the approach with various oils, surfactants, and co-surfactants to identify which one has the best solubilizing capacity for the drug under investigation and to load the drug to produce the best results [6,23,24].

Lemongrass oil was chosen as it has antimicrobial, antioxidant, antifungal, and anti-inflammatory effects [25,26]. In addition, lemongrass oil also gives the formula a good smell, which increases the product’s acceptability. Lemongrass oil can specifically solubilize the lipophilic drug by increasing the transport fraction and thus increasing absorption and penetration. These properties of lemongrass oil refer to the presence of monoterpenes and sesquiterpenes, which are known as penetration enhancers and skin protectants [27].

The surfactants used in the formula were safe, biocompatible, and non-ionic hydrophilic [28,29]. Furthermore, these surfactants give the nanoemulsion rapid drug release and absorption because of their ability to form droplets that have a large surface area. The hydrophilic-lipophilic balance (HLB) value of Tween 80 is 15, and for Span 80 is 4.3. A system with a required HLB value was created by mixing different concentrations of them to get an O\W emulsion easily [30].

In the self-nano emulsifying drug delivery system (SNEDDS), different formulations with varying concentrations of lemongrass oil, Tween 80, and Span 80 were tested, and the ternary phase diagram was employed to choose the formulation that produced the smallest particles, less than 200 nm in size. The amount and rate of drug release and absorption were governed by two crucial factors: particle size and PDI. The SNEDDS criteria were met when the droplet size was less than 200 nm and the polydispersity value was near zero.

The droplet size was crucial to the success of SNEEDS since it controlled the quantity and rate of drug release and absorption [31]. Moreover, the reduced particle size and increased interfacial surface area led to quicker absorption and enhanced bioavailability [32,33]. The SNEDDS criterion was accomplished when the size of the droplets was smaller than 200 nm. As previously stated, the correct surfactant/co-surfactant combination resulted in a smaller globule size and a strong mechanical barrier that prevented aggregation and coalescence of the generated globule. [34,35].

Another important factor in the formulation of SNEEDS is PDI. It is also called the droplet size distribution, which measures particle homogeneity. When the PDI is near zero, the particles are more homogeneous and form a more uniform emulsion, which has higher physical stability [36]. A colloidal preparation’s stability is determined by the value of its zeta potential. If the zeta potential of the particles is mostly negative or positive, they will repel one another, leading to a stable dispersion. If the values are low, there will be dispersion instability because the particles will clump. The dividing line between stable and unstable dispersions is often determined at +30 or −30 mV [37,38]. Particles were thought to be stable when their zeta potential was either 30 mV or less, making any of these values acceptable [39]. All BPO nanoemulgel formulations exhibited a zeta potential for the drug lower than −35 mV owing to the presence of non-ionic surfactants, which stabilize the system by forming a coating over the surface of the nanoparticles [40]. They do not contribute to any change in the nanoemulsion particle. Hence, it has no effect on the nanoemulsion’s stability [41].

Rheological measurements are essential for semi-solid formulations because they enable us to physically define the system (flow characteristics) and manage its stability, which is essential for assuring the formulation’s efficacy and quality. Higher viscosities may alter drug release and bioavailability by delaying drug diffusion out of the vehicle, making the drug less bioavailable [42]. According to the data, when the concentration of Carbopol increased, the viscosity increased while the shear rate reduced. The pseudo-plastic behavior of the colloidal-rheological emulgel indicates that viscosity decreases as the shear rate increases.

The rapid release of the drug into the dissolution medium characterizes the delivery system’s efficiency [43,44]. The release of the drug decreases with each addition of Carbopol due to the increase in viscosity. As a result, the formulation containing 0.4% Carbopol will have the best pattern of drug release when compared to the other formulations in the study (0.6%, 0.8%, and 1%) and the market product. These findings were obtained in 2007 by Malay K. Das and Abdul B. Ahmed, who observed that there is an inverse relationship between Rofecoxib release rate and the concentration of Carbopol [45].

BPO nanoemulgels demonstrated improved antibacterial activity and a larger zone of inhibition compared to the commercial drug. Several factors have contributed to these results. First, because the nanoparticles have a small nano-size and a larger surface area, they have greater penetration and activity [46]. The same results were proven by Qurat-ul-Ain Naqvi et al. (2019), who showed that the smaller size of ZnO particles has more bacterial activity inhibition [47].

In addition, the nanoparticles in the nanoemulsion-based hydrogel increase the concentration of the drug that penetrates the bacteria because they increase the contact time between them [48,49]. The inclusion of lemongrass oil, which exhibited antimicrobial action against gram-negative, gram-positive, and acne bacteria, was the second reason for the improvement in bacterial inhibition, which led to the development of the drug nanoemulgels [50,51].

## 4. Conclusions

Benzoyl peroxide nanoemulgels were successfully prepared in this research by developing a nanoemulsion of lemongrass oil, Tween 80, and Span 80. A self-nano emulsifying technique was used to create the nanoemulsion, which was then combined with Carbopol hydrogel to produce a nanoemulgel. These nanoemulgels were found to have high stability, as indicated by the zeta potential and similar rheological behavior. When compared to the market product, 0.4% Carbopol has the highest drug release and antimicrobial activity. Overall, the study’s findings indicated that the BPO nanoemulgel could be a promising dosage form for pharmaceutical industries.

## 5. Materials and Methods

### 5.1. Materials

Benzoyl peroxide was purchased from Aldrich, USA, Benzac AC (Market product of benzoyl peroxide), Polysorbate 20 (Tween 20), Sorbitan laurate (Span 20), Polysorbate 80 (Tween 80), Sorbitan oleate (Span 80), Polyacrylic acid polymer (Carbopol 940) were purchased from CBC Co., Ltd., Tokyo, Japan. Lemongrass oil, olive oil, castor oil, crystal oil, paraffin, corn oil and coriander oil, Rosemary oil and propylene glycol were obtained from the AL-Shams company, Jifna, Palestine.

### 5.2. Determination of Wavelength and Calibration Curve of Benzoyl Peroxide

In order to scan the lambda max for BPO, 10 mL of ethanol was used with 0.02 g of the active ingredient (BPO). The vortex mixer was used to mix the formulation in order to confirm the homogeneity of the sample. The UV spectrophotometer (Jenway 7315-spectrophotometer, Dunmow, UK) was used to determine the absorbance at a wavelength range of 200–400 nm.

### 5.3. Solubility Analysis of Benzoyl Peroxide in Different Oils and Surfactants

The dissolving of the BPO in various oils (Lemongrass oil, olive oil, castor oil, crystal oil, paraffin, corn oil, coriander oil, Rosemary oil, and propylene glycol) and surfactants (Tween 20, Tween 80, Span 20, and Span 80) to choose the best oil and surfactant that has the highest solubility. The samples were put in an isothermal shaker (Nirmal International, Delhi, India) at 25 ± 1.0 °C until equilibrium was achieved. After 72 h, the supernatant was pulled from each sample and filtered through a membrane filter (0.22 μm), then it was put into a centrifuge at 3000 rpm for 15 min [6]. The absorption was determined for the drug in each sample using a UV spectrophotometer.

### 5.4. Preparation of Lemongrass Oil Nanoemulsion

A self-nanoemulsifying technique was used to prepare the nanoemulsion. Depending on the solubility testing of different oils and surfactants, the selected lemongrass oil was mixed with Span 80 and Tween 80 in a different ratio to build up a ternary phase diagram. Then the formulations were measured for the droplet size and polydispersity index (PDI) using Omni Brookhaven (Brookhaven Instruments Corporation, Holtsville, NY, USA). This was done by taking one drop from each mixture and self-emulsifying it in 60 mL of purified water with gentle agitation to produce the nanoemulsion. The best formulation was chosen based on the measurement of the droplet size, which should be below 200 nm, the PDI below 0.3 and the oil concentration [52].

### 5.5. Loading Benzoyl Peroxide into the Nanoemulsion Formulation

After choosing the optimum nanoemulsion formulation, it was used to load the BPO in the formulation, and then it was sonicated using (Elmasonic S100H, Singen am Hohentwiel, Gemrany) for one hour to achieve homogeneity. The particle size and PDI were measured again in triplicate.

### 5.6. Hydrogel Preparation

The hydrogel was made by dissolving Carbopol 940 in water and continuously stirring it with a homogenizer to achieve uniform dispersion. By adding 2 M NaOH while stirring, the pH of the hydrogel was changed to 6. It was left to gel for 24 h, at which point it was ready.

### 5.7. Nanoemulgel Preparation of Benzoyl Peroxide

The selected nanoemulsion formulation, loaded with the drug, was incorporated with water and shacked well, then Carbopol hydrogel was added in different concentrations (0.4%, 0.6%, 0.8%, and 1%) and mixed by using a glass rod. The resultant nanoemulgel was subjected to particle size and PDI analysis.

### 5.8. Rheological Measurement for Benzoyl Peroxide Nanoemulgel

Different concentrations of Carbopol as a thickening agent cause some differences in the behavior of nanoemulgel formulations. At the same time, measurements were taken using a rotational viscometer at the same temperature of 25 °C (Brookfield DVI, Middleboro, MA, USA). The viscosity values were in the 0–100 rpm shear rate range [53].

### 5.9. Release Test of Benzoyl Peroxide from Different Nanoemulgel Formulations

A 1.5 mL sample of each nanoemulgel formulation with a different Carbopol concentration (0.4%, 0.6%, 0.8%, and 1%) was loaded into a dialysis bag (Spectra/pro 3 Dialysis membrane, P/N 132 700) with a 16 mm diameter. Phosphate buffer saline (1X, PH 7.4) was prepared by dissolving 8 g of NaCl, 200 mg of KCl, 1.44 g of Na_2_HPO_4_ and 245 mg of KH_2_PO_4_. Distilled water was added until the volume was 800 mL, then PH was adjusted to 7.4. The dialysis bag, which was loaded with the nanoemulgel formulations, was added to 40 mL of the buffer in the isothermal shaker (Nirmal International, Delhi, India) at 37 ± 1 °C. The samples were taken from the buffer solution at 10, 20, 30, 60, and 120 min to determine the release of the drug from the nanoemulgel by measuring absorbance using a spectrophotometer. The release test was also performed on the market product (Benzac 5%) in order to compare with our formulations’ release [6].

### 5.10. Antibacterial Test

#### 5.10.1. Microorganisms

The organisms used for the antibacterial test were Methicillin-Resistant *Staphylococcus Aureus* (MRSA), *E. coli*, *Pseudomonas Aeruginosa*, *Klebsiella Pneumonia*, *Staphylococcus Aureus*, *Candida*, and *Proteus Mirabilis*.

#### 5.10.2. Culture Media

The culture medium was molar Hinton agar. It was prepared by mixing 2 g of beef extract, 1.5 g of starch, 17.5 g of acid hydrolysate, and 17 g of agar per liter of purified water. The components were mixed together and heated until boiling, with continuous stirring. The mixture was placed in the autoclave for 20 min at 121 °C. Then, the mixture was cooled and distributed into sterile Petri dishes.

Six Petri dishes were cultured with previous types of bacteria. Then, four holes (A, B, C, D) were made in each dish. A was filled with market drug (Benzac), B was filled with nanoemulgel that contained 0.4% Carpobol but without the drug, C was filled with nanoemulgel that contained 0.4% Carpobol but with the drug, and D was filled with lemongrass oil. The dishes were incubated at 37 °C. After 24 h, the diameter of the zone of inhibition was measured to determine the antibacterial activity of our samples [53].

### 5.11. Anti-Acne Test

#### 5.11.1. Microorganism

The organism used for the anti-acne test was *Cutibacterium acne*.

#### 5.11.2. Culture Media

The culture media used for the test was Trypticase Soy agar broth with Defibrinated Sheep Blood and Modified Reinforced Clostridial. The bacteria came as a powder in vials. The vials were opened and dissolved with 0.5 ml of broth media (Modified Reinforced Clostridial) under an anaerobic condition. Then the entire contents were transferred to 5–6 mL of broth. The agar media was cultured with the previous bacterial-rich broth media. The agar media was incubated under an anaerobic condition at 37 °C for 48–72 h, then two holes (A and B) were made in the agar media. A was filled with nanoemulgel formulation, and B was filled with the market product (Benzac) [54].

### 5.12. Statistical Assessment

All of the established experiments’ results were collected in triplicate, and the values were expressed as mean ± S.D. When the *p*-value was 0.005, statistical significance was considered.

## Figures and Tables

**Table 1:** Solubility of Benzoyl peroxide in different oils and surfactants.

Oils	Concentration (mg\mL)
Crystal oil	2.3
Propylene glycol	3.2
Castor oil	6.02
Corn oil	7.76
Lemongrass oil	8.9
Paraffin oil	7.1
Coriander oil	8.6
Olive oil	7.6
Rosemary oil	7.04
Tween 20	5.76
Tween 80	6.27
Span 20	3.71
Span 80	6.2

**Table 2:** The antibacterial activity of lemongrass oil, lemongrass nanoemulgel and Benzoyl peroxide nanoemulgel compared with the market product.

Bacteria	Market Product	BPO Nanoemulgel	Lemongrass Oil Nanoemulgel	Lemongrass Oil (Pure Oil)
MRSA	16 ± 0.8	44 ± 1.5	30 ± 1.1	28 ± 0.4
*E. coli*	R	R	R	R
*Pseudomonas*	R	44 ± 1.3	35 ± 0.2	28 ± 0.7
*Klebsiella*	R	R	R	R
*Staphylococcus Aureus*	12 ± 0.6	23 ± 0.9	20 ± 0.8	19 ± 1.1
Proteus	12 ± 0.7	49 ± 1.2	42 ± 1.1	30 ± 0.9
*Candida*	R	36 ± 1.0	30	26 ± 1.0

**Table 3:** The ant-acne activity of BPO nanoemulgel compared with the market product.

Bacteria	Market Product	BPO Nanoemulgel	Lemongrass Oil Nanoemulgel	Lemongrass Oil (Pure Oil)
*Cutibacterium acne*	38 ± 0.8	50 ± 1.2	36 ± 1.3	27 ± 0.9

## Data Availability

All data are included in the manuscript.
